# Quercetin and Quercitrin Attenuates the Inflammatory Response and Oxidative Stress in LPS-Induced RAW264.7 Cells: In Vitro Assessment and a Theoretical Model

**DOI:** 10.1155/2019/7039802

**Published:** 2019-10-28

**Authors:** Jie Tang, Ping Diao, Xiaohong Shu, Li Li, Lidan Xiong

**Affiliations:** ^1^Cosmetics Safety and Efficacy Evaluation Center, West China Hospital, Sichuan University, No. 5, Gong Xing Road, Chengdu, Sichuan 610041, China; ^2^Sichuan Engineering Technology Research Center of Cosmetic, Chengdu, Sichuan 610041, China; ^3^Department of Dermatology, West China Hospital, Sichuan University, No. 37, Guo Xue Xiang, Chengdu, Sichuan 610041, China

## Abstract

**Background:**

Nowadays, atmospheric pollutants, ultraviolet rays, and other factors cause the imbalance of cell redox, resulting in skin oxidative damage. There is an interaction between inflammatory response and oxidative stress, which often involve networks of reactions and serve to amplify each other. Quercetin and quercitrin, with strong antioxidant and anti-inflammatory properties, were widely applied in cardiovascular disease, osteoporsis, pulmonary disease, etc. However, the regulation mechanism of quercetin and quercitrin on various inflammatory skin diseases is still not clear.

**Purpose:**

In this study, quercetin and quercitrin were used to investigate whether they had anti-inflammatory and anti-ROS effects. Besides, theoretical calculation method was also adopted to preliminarily explore the mechanism of the anti-inflammatory and antioxidant effects of these two substances.

**Methods:**

CCK-8 assay was employed to investigate the cytotoxicity. The concentration of NO measured by Griess Reaction System. Moreover, the inflammatory factors (TNF-*α*, IL-1*β*, and IL-6) were reduced in LPS-stimulated RAW264.7 cells were tested by ELISA kits. The trend of ROS changes was detected by DCFH-DA method. Finally, the mechanism of the anti-inflammatory and antioxidant effects of these two substances was carried out by DMol3 package in Materials Studio.

**Results:**

CCK-8 assay results guided that the safe concentration of quercetin and quercitrin was lower than 15.0 *μ*g/mL and 22.4 *μ*g/mL, respectively. Also, the concentration of NO could significantly be inhibited by quercetin and quercitrin. Besides, the ELISA results showed that TNF-*α*, IL-1*β*, and IL-6 were reduced in LPS-stimulated RAW264.7 cells after interfering with quercetin and quercitrin. The trend of ROS changes was similar to that of inflammatory factors. Finally, the theoretical calculation illustrated that the oxygen atom on B rings may be the main site of electron cloud density changes, which may suggest a possible mechanism for the anti-inflammatory and ROS scavenging effects of quercetin and quercitrin.

**Conclusions:**

This experiment shows that LPS can induce the overactivating of macrophages and the activated macrophages can subsequently induce inflammatory storms and oxidative stress. Both quercetin and quercitrin can inhibit LPS-induced macrophage inflammation and oxidative stress by experiment and theoretical calculations.

## 1. Introduction

Oxidative stress refers to the imbalance of oxidation and antioxidation in the body under the attack of harmful stimulating factors [1]. A large amount of reactive oxygen species (ROS) directly or indirectly cause oxidative damage to cells [2, 3], leading to an inflammation reaction where the local tissue is mainly resistant to invasion by chemical or biological factors. As the outermost immune organ of the human body, the skin is susceptible to oxidative stress caused by environmental imbalance, which causes various skin damage and diseases, such as aging, tumor, and inflammation. In the face of ultraviolet light, air pollution, and other external factors on the skin, it is of great significance to use substance that can inhibit oxidative stress and inflammatory reaction to treat inflammatory skin lesions and slow down skin aging.

**Figure 1:**
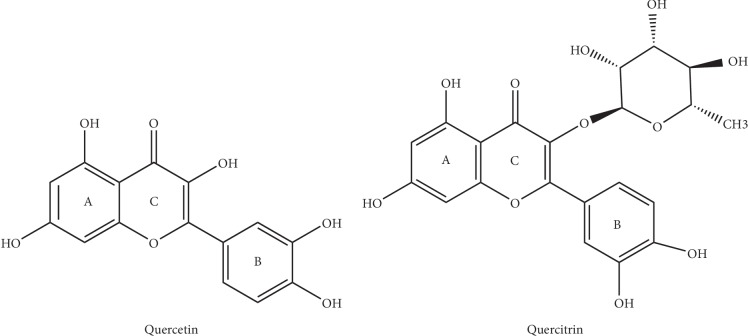
The molecular structure formula of quercetin and quercitrin.

Quercetin (4H-1-benzopyran-4-one, 2-(3,4-dihydroxyphenyl)-3,5,7-trihydroxy-flavon) and quercitrin (2-(3,4-dihydroxyphenyl)-5,7-dihydroxy-4-oxo-4H-chromen-3-yl6-deoxy-alpha-L-mannopyranoside) are a class of natural flavonoids widely found in the flowers, leaves, and fruits of various plants. They have anticancer, antifibrosis, anti-inflammatory, and antioxidation effects [1, 4, 5]. Both quercetin and quercitrin are biological flavonoids, and their structures are similar. They mainly refer to two benzene rings (A ring and B ring) as the mother nucleus, which are linked by a central three carbon chain (C chain). The series of compounds with the C6-C3-C6 basic carbon skeleton contain a plurality of hydroxyl groups (OH) and are highly polar [6, 7]. The basic structure is shown in Figure 1. It can be speculated from the structure that it has the ability to scavenge free radicals, but the position of the two molecules on the B ring is different, which affects its anti-inflammatory activities and antioxidation effects.

In this study, mouse mononuclear macrophage leukemia cells (RAW264.7) were used as the inflammatory model induced by lipopolysaccharide (LPS) stimulation. The cytotoxicity, ROS, nitric oxide (NO), and the inflammatory factors tumor necrosis factor (TNF-*α*), interleukin-1*β* (IL-1*β*), interleukin-6 (IL-6) of RAW264.7 regulated by quercetin and quercitrin were investigated. Finally, a theoretical calculation was adopted to explore the antioxidant capacity of OH on quercetin and quercitrin at different positions, providing a foundation for anti-inflammatory and antioxidation research.

## 2. Experimental

### 2.1. Materials and Instrument

RAW264.7 were purchased from Kunming Institute of Zoology, Chinese Academy of Science. Dulbecco's Minimum Essential Medium (DMEM) culture medium, penicillin streptomycin combination, and trypsin (0.25%) was acquired from HyClone (GE Health Care Life Science, Little Chalfont, Buckinghamshire, UK). Fetal bovine serum (FBS) was gained from Gibco (Thermo Fisher, Waltham, MA, USA). Phosphate buffers solution (PBS 10x) was purchased from Zsbio Commerce CO (Zsbio, China). NO kits were acquired from Beyotime® Biotechnology (Beyotime, Shanghai, China). TNF-*α*, IL-1*β*, and IL-6 kits were obtained from Boster® Biotechnology (Boster, Wuhan, China). LPS, quercetin, quercitrin, 2′,7′-dichlorofluorescin (DCFH-DA), and dexamethasone (DEX) were purchased from Sigma-Aldrich (St. Louis, MO, USA). Cell Counting Kit-8 (CCK-8) was obtained from Dojindo (Dojindo Laboratories, Kumamoto, Japan).

RAW264.7 cells culture medium was supplemented with 10% fetal bovine serum and 100 U/mL penicillin and 100 *μ*g/mL streptomycin in DMEM culture medium, and then incubated in 5% CO_2_ incubator within a humidified atmosphere at 37°C. Microplate spectrophotometer was gained from Bio-Rad (Bio-Rad Inc, Hercules, CA, USA). The pictures were visualized by an inverted fluorescence microscope (Olympus Opticals, Tokyo, Japan).

### 2.2. Cell Viability

The cytotoxicity of quercetin and quercitrin on RAW264.7 cells were assessed by CCK-8 assay. Briefly, cells dispersed evenly in medium were seeded at a density of 4.0 × 10^4^ cells/well in 24-well plates. Next day, cells were treated with quercetin (0.03 *μ*g/mL to 15 *μ*g/mL) and quercitrin (0.045 *μ*g/mL to 22.4 *μ*g/mL), respectively, for 24 hours. Then, CCK-8 solution was added into each well. Followed by a 4-hour incubation, the optical density (OD) at 450 nm was determined.

### 2.3. Assessment of NO Production

The RAW264.7 cells in the logarithmic growth phase were inoculated into a 24-well plate at 4.0 × 10^4^ cells/well for 24 hours. Then, the experiment was divided into control group, LPS + PBS (model group, LPS: 2 *μ*g/mL), LPS + DEX (positive control group, LPS: 2 *μ*g/mL, DEX: 10 *μ*g/mL), LPS + LQCT (LPS: 2 *μ*g/mL, quercetin: 0.03 *μ*g/mL), LPS + MQCT (LPS: 2 *μ*g/mL, quercetin: 0.3 *μ*g/mL), LPS + HQCT (LPS: 2 *μ*g/mL, quercetin: 3 *μ*g/mL), LPS + LQTR (LPS: 2 *μ*g/mL, quercitrin: 0.045 *μ*g/mL), LPS + MQTR (LPS: 2 *μ*g/mL, quercitrin: 0.45 *μ*g/mL), LPS + HQTR (LPS: 2 *μ*g/mL, quercitrin: 4.5 *μ*g/mL) groups. In order to determine the effect of quercetin and quercitrin on NO production, the amount of NO in the supernatant was detected using a commercially available NO detection kit. Briefly, equal volumes of Griess Reagent I and Griess Reagent II were added, and the absorbance at 540 nm was detected by a microplate reader. The NO content was calculated from a nitrite standard curve.

### 2.4. Assessment of TNF-*α*, IL-1*β*, and IL-6 Using ELISA Kits

RAW264.7 cells were seeded in 6-well plate at 1.0-2.0 × 10^5^ cells/well for 24 hours. After that, the cells were treated as well as Section 2.3. The culture supernatant was collected and centrifuged at 3000 rpm for 20 min. Finally, the cellular abundances of TNF-*α*, IL-1*β*, and IL-6 were detected in the medium by corresponding ELISA kit according to the manufacturer's instructions.

### 2.5. Assessment of ROS

The accumulation of ROS was assessed by the radical probe DCFH-DA. At the beginning, RAW264.7 cells were seeded in 6-well plate at 1.0∼2.0 × 10^5^ cells/well for 24 hours and were treated the same as Section 2.3. And then, 1 mL DCFH-DA diluted with 1 : 1000 in serum-free medium was added after removing all the cell culture medium. After incubated at 37°C for 20 minutes in the dark, the cells were washed 3 times with PBS to sufficiently remove DCFH-DA that had not been entered into the cells. Thereafter, the cells were visualized by an inverted fluorescence microscope. After observation, the cells were collected and detected by a fluorescent microplate reader.

### 2.6. Theoretical Calculation of Antioxidant Model

All density functional theory (DFT) calculations were performed with DMol3 [1] package in Materials Studio as the measure of theoretical calculation to achieve structural optimization [8, 9]. PW91 of correlation function of generalized gradient approximation (GGA) level was conducted [10]. The valence electron wave function was developed by a double numerical plus polarization function (DNP), which was equivalent to 6-31G^*∗∗*^ in Gaussian. All atoms were all-electron basis sets, and the structure optimization was based on the convergence of energy, displacement, and force convergence, which convergence threshold valve is set to 2 × 10^−5^ Hartree, 4 × 10^−3^ Hartree/Å and 5 × 10^−3^ Å, respectively. Self-consistent field (SCF) calculation was carried out with a tolerance of 1 × 10^−5^ a.u., and the smearing value associated with the thermal occupancy effect was selected to be 0.005 Hartree. Conductor-like screening model (COSMO) solvent calculation with the DC-PBE functional was also performed using the dielectric constant of ethanol (24.3).

### 2.7. Statistical Analysis

Data analysis was performed by using the SPSS17.0 software (SPSS for Windows, Chicago, SPSS Inc.). The experiment was repeated 3 times and the data were expressed as mean ± standard deviation ($\bar{x}$ ± *s*). Variances of data were compared by using variance analysis, differences between the two groups were compared by *t*-test, and it was considered statistically significant if the *P* value was equal to or less than 0.05.

## 3. Results

### 3.1. Cytotoxicity of Quercetin and Quercitrin on RAW264.7 Cells

**Figure 2:**
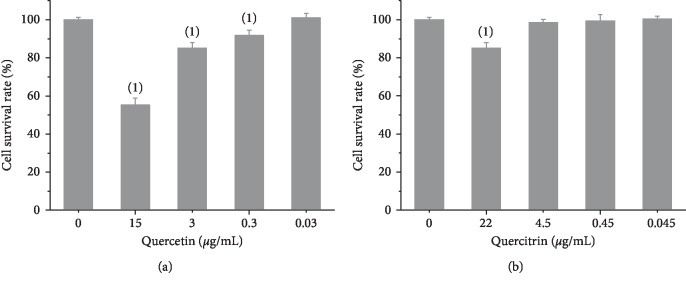
The cell survival rate of quercetin (a) and quercitrin (b) on RAW264.7 cells. The results represent the $\bar{x}$ ± *s* (*n* = 3). ^(1)^*P* < 0.01; ^(2)^*P* < 0.05, compared with control group.

In order to ascertain whether quercetin and quercitrin exerted cytotoxicity on RAW264.7 cells, the CCK-8 assay was performed. As shown in Figure 2, the treatment with quercetin (15.0 *μ*g/mL) and quercitrin (22.4 *μ*g/mL) for 24 hours significantly decreased the cell viability compared with the control group. Besides, in order to study the anti-inflammatory effects of quercetin and quercitrin, we chose these three concentrations (3.0, 0.3, 0.03 *μ*g/mL for quercetin; 4.5, 0.45, 0.045 *μ*g/mL for quercitrin) for further investigation.

### 3.2. Effects of Quercetin and Quercitrin on the Production of NO in LPS-Induced RAW264.7 Cells

**Figure 3:**
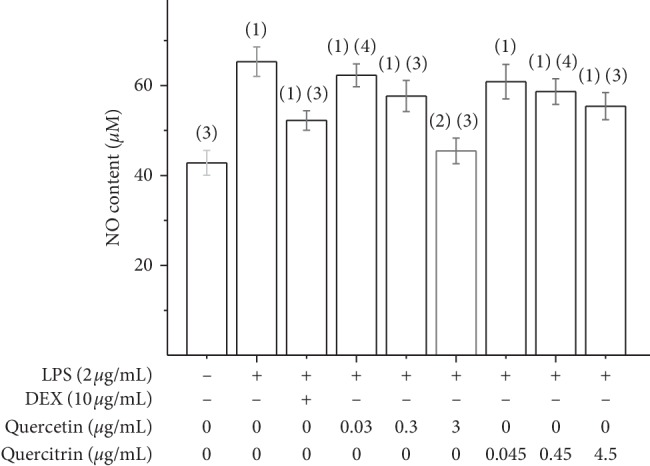
The effect of quercetin and quercitrin of NO content on RAW264.7 cells induced by LPS, $\bar{x}$ ± s (*n* = 3). ^(1)^*P* < 0.01; ^(2)^*P* < 0.05, compared with control group; ^(3)^*P* < 0.01; ^(4)^*P* < 0.05, compared with LPS group.

The RAW264.7 cells were exposed to various concentrations of quercetin and quercitrin and induced with LPS (2 *μ*g/ml). The NO concentrations are shown in Figure 3. The amount of NO released by RAW264.7 cells increased significantly when exposed to LPS, and the treatment with positive control (DEX, 10 *μ*g/mL), quercetin, and quercitrin significantly decreased the LPS-induced NO production in RAW264.7 cells. Briefly, treatment with quercetin and quercitrin after the LPS activation could significantly suppress the secretion of NO. At low concentrations, the two substances had similar inhibitory effects on NO, but when the concentration rose high (3 *μ*g/mL for quercetin, 4.5 *μ*g/mL for quercetrin), quercetin had stronger inhibitory effect on NO production than quercitrin.

### 3.3. Effects of Quercetin and Quercitrin on TNF-*α*, IL-1*β*, and IL-6 Induced by LPS

**Figure 4:**
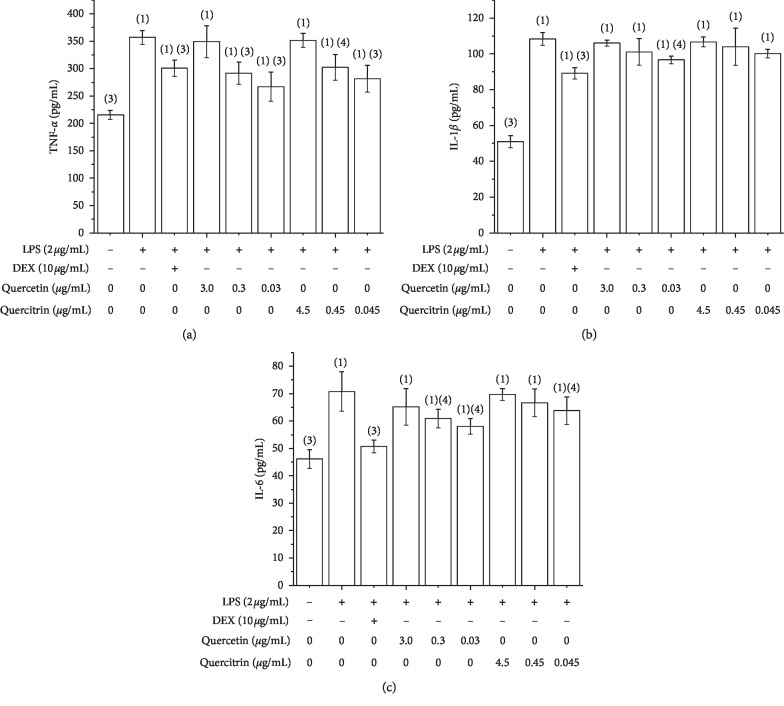
The effect of quercetin and quercitrin of TNF-*α* (a), IL-1*β* (b), and IL-6 (c) contents on RAW264.7 cells induced by LPS, $\bar{x}$ ± *s* (*n* = 3). ^(1)^*P* < 0.01; ^(2)^*P* < 0.05, compared with control group; ^(3)^*P* < 0.01; ^(4)^*P* < 0.05, compared with LPS group.

To investigate the inhibitory effect of quercetin and quercitrin on TNF-*α*, IL-1*β* and IL-6 production, the cells were treated with LPS in the absence or presence of quercetin and quercitrin, and the levels were measured by ELISA. As shown in Figure 4, compared with unstimulated cells, the level of TNF-*α*, IL-1*β*, and IL-6 were increased in LPS-induced cells. After LPS-induced cells, positive control (DEX), quercetin, and quercitrin were added. Compared with LPS group, the level of TNF-*α*, IL-1*β*, and IL-6 decreased significantly in the DEX, quercetin, and quercitrin groups.

### 3.4. Effects of Quercetin and Quercitrin on ROS Levels Induced by LPS

**Figure 5:**
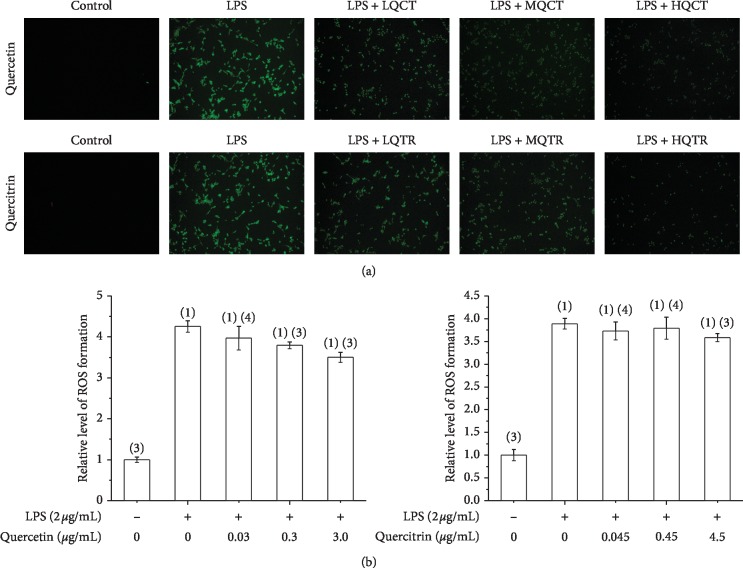
(a): Effects of quercetin and quercitrin on LPS-induced RAW246.7 cells detected by DCFH-DA assay (×100); (b) effects of quercetin and quercitrin on ROS, $\bar{x}$ ± *s* (*n* = 3). ^(1)^*P* < 0.01; ^(2)^*P* < 0.05, compared with control group; ^(3)^*P* < 0.01; ^(4)^*P* < 0.05, compared with LPS group.

To determine whether quercetin and quercitrin can scavenge the ROS, the DCFH-DA assay was carried out. As shown in Figure 5, LPS treatment induced ROS accumulation compared to untreated cells. Quercetin and quercitrin reduced the LPS-stimulated elevation of ROS production, and the fluorescence intensity was significantly decreased in a dose-dependent manner.

### 3.5. Superficial Mechanism of the Antioxidant Properties of Quercetin and Quercitrin

In order to further illustrate the anti-inflammatory and anti-ROS mechanism of quercetin and quercitrin, we used the theoretical calculation. The results are shown in Table 1. Firstly, the optimization structures of quercetin and quercitrin were presented. Then, the highest occupied molecular orbital (HOMO) and lowest unoccupied molecular orbital (LUMO) were shown. The oxygen atom (O) belonging to OH on the B rings of quercetin and quercitrin had obvious electron cloud changes in both HOMO and LUMO orbitals, which indicated that the “target” of the antioxidant reaction may be the lone pair of electrons on the oxygen atom. In addition, we calculated the HOMO-LUMO gap (H-L gap), which was important for molecules since they indicated their chemical stability in various states such as reduced and oxidized [11, 12]. The higher the H-L gap values are, more stable the chemical stabilities are, and more difficultly the reaction happens. The results showed that the gap value of quercetin (2.312 eV) was less than that of quercitrin (2.577 eV), which suggested that quercetin had slightly higher antioxidant and anti-inflammatory properties than quercitrin to some extent.

## 4. Discussion

**Figure 6:**
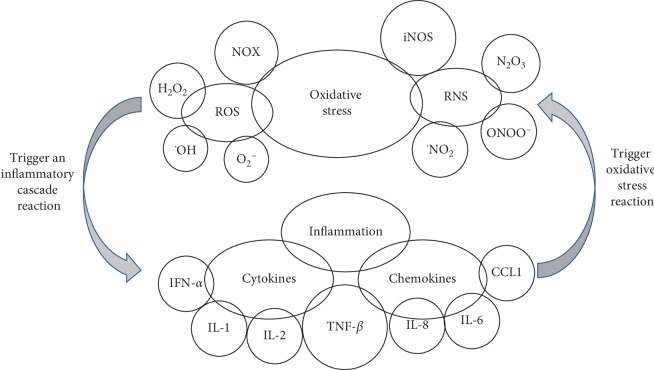
Interrelation of oxidative stress and inflammation.

Macrophages are one of the most important cells in the skin immune system, and they are also the key cells to regulate the healing of wounded skin [13]. In wound healing, macrophages secrete proinflammatory factors to initiate inflammation firstly. Then, resolve inflammation by secreting anti-inflammatory factors and phagocytizing apoptotic cells. At the same time, macrophages secrete cytokines, growth factors, and chemokines to promote wound epithelial regeneration, collagen deposition and angiogenesis [14, 15]. At present, the regulation mechanism of macrophages on various inflammatory skin diseases is still not clear. Besides, atmospheric pollutants, ultraviolet rays, and other factors cause the imbalance of cell redox. Excessive ROS activates inflammatory cells (such as macrophages, lymphocytes, and neutrophils), inducing inflammatory as a result. There is an interaction between inflammatory response and oxidative stress. On one hand, inflammatory cells produce ROS which participates in oxidative stress; on the other hand, ROS can lead to increasing expression of inflammatory cytokines [2, 3]. Besides, ROS can be used as the second messenger downstream of some special ligands (TGF-beta, EGF-2, PDGF, etc.) to participate in the regulation of intracellular inflammatory signal transduction pathway, and it also regulates the activity of some inflammatory transcription factors (such as NF-*κ*B). A large number of oxidative intermediates lead to inflammatory cell infiltration, and the activated immune cells secrete a series of bioactive substances, such as protease and ROS mediators, which can lead to tissue damage [2]. Meanwhile, immune cells also have the functions of phagocytosis, clearance of ROS, antigen presentation, and secretion of a variety of cytokines. In short, oxidative stress and inflammation can amplify each other's role and accelerate skin tissue damage and lesions. So the role of oxidative stress in inflammatory skin diseases has attracted increasing attention. The association between inflammation and oxidative stress is shown in Figure 6.

This experiment shows that LPS can induce the over-activating of macrophages and the activate macrophages can subsequently induce inflammatory storms and oxidative stress. Both quercetin and quercitrin can inhibit LPS-induced macrophage inflammation and oxidative stress by experiment and theoretical calculations.

A large amount of NO produced by LPS is related to inflammatory reaction. Furthermore, NO can interact with other radicals to generate cytotoxic molecules. Hence, inhibition of NO production is an anti-inflammatory treatment. In this study, treatment with quercetin and quercitrin after the LPS activation could significantly suppress the secretion of NO. Besides, ROS is an important molecular target of inflammatory disease. Excessive production of ROS in LPS-induced macrophages can cause proinflammatory response by upregulating proinflammatory cytokines and functioning as a secondary messenger in following progression [16]. In our study, quercetin and quercitrin did scavenge the ROS production in macrophages. Compared with LPS treatment group, quercetin and quercitrin markedly downregulated TNF-*α*, IL-1*β*, and IL-6.

According to the articles, the main mechanism is that phenolic OH reacts with free radicals to form semiquinone free radicals which can terminate the free radical chain reaction [16]. It can be seen from Table 1 that the antioxidant capacity is related to the electron cloud changes in the aromatic ring of quercetin and quercitrin. When the reaction between the substance and the free radical loses electrons or supplies hydrogen, a new group will be formed, which is stabilized by the spin action of the aromatic nucleus, and the oxidation reaction of the substance will be interrupted or delayed [17–19]. Therefore, the antioxidant ability of quercetin and quercitrin is positively related to the stability of the group formed. Previous studies have shown that B-ring in the class of natural flavonoids is the main active site for antioxidant and ROS scavenging. The theoretical calculation of this experiment is the same as previous studies. The ROS scavenging activity is directly related to the number and location of OH on B-ring. However, the difference of hydroxyl radical scavenging ability between different sites needs to be further optimized.

## Figures and Tables

**Table 1:** The optimization of configuration parameters and molecular frontier orbit properties of quercetin and quercitrin.

Substance	Structural formula	HOMO	LUMO	H-L gap (eV)
Quercetin	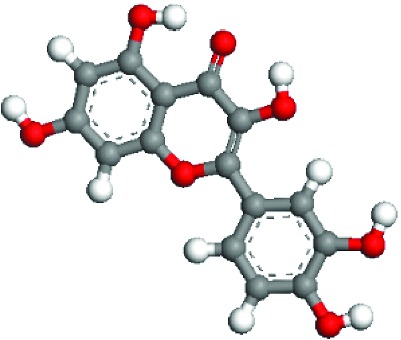	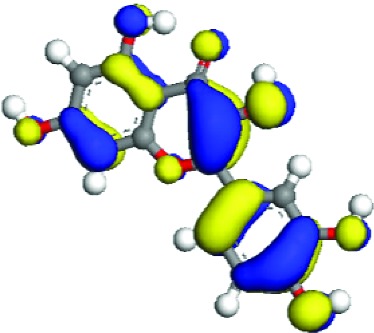	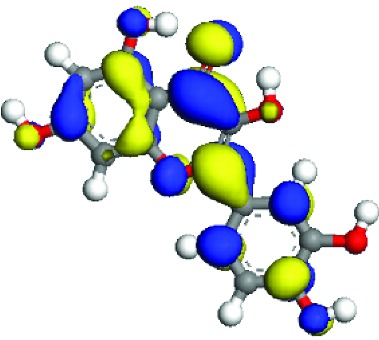	2.312
Quercitrin	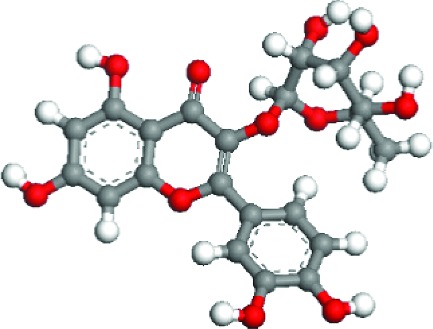	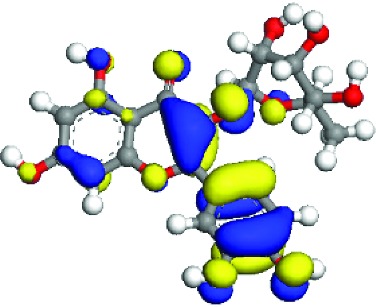	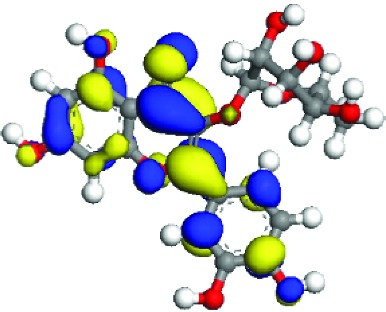	2.577

## Data Availability

The original data used to support the findings of this study are available from the corresponding author upon request.
